# Optimizing N Fertilization to Improve Yield, Technological and Nutritional Quality of Tomato Grown in High Fertility Soil Conditions

**DOI:** 10.3390/plants9050575

**Published:** 2020-05-01

**Authors:** Domenico Ronga, Alfonso Pentangelo, Mario Parisi

**Affiliations:** 1 Department of Life Science, University of Modena and Reggio Emilia, Via Amendola, n. 2, 42122 Reggio Emilia, Italy; domenico.ronga@unimore.it; 2 CRPA Centro Ricerche Produzioni Animali, viale Timavo 43/2, 42121 Reggio Emilia, Italy; 3 CREA Research Centre for Vegetable and Ornamental Crops, Via Cavalleggeri, 25, 84098 Pontecagnano Faiano, Italy; alfonso.pentangelo@crea.gov.it

**Keywords:** *Solanum lycopersicum* L., sustainability, harvest index, N-efficiency, Brix, nitrate, mineral composition

## Abstract

Processing tomato is the second most important worldwide cash crop, generally produced in high-input systems. However, fruit yield and quality are affected by agronomic management, particularly nitrogen (N) fertilization, whose application to indeterminate growth genotypes for canning has yet to be investigated in depth. Hence, the objective of this work was to assess the effects of different N rates (0, 50, 125, 200, 275, and 350 kg ha^−1^) on fruit yield and quality characteristics of processing tomato ‘San Marzano’ landrace. The results of our study showed that 125 and 200 kg of N ha^−1^ are the most appropriate rates in soil with high fertility, ensuring the highest values of marketable yield and brix yield. However, plants fertilized with 125 kg of N ha^−1^ attained higher values of N efficiency and fruit K and P concentrations than plants fertilized with 200 kg of N ha^−1^. Our results suggest that overdoses of N supplies negatively affected fruit yield and quality of San Marzano landrace grown in high soil fertility conditions, also reducing the agricultural sustainability. Hence, specific agronomic protocol and extension services are required to optimally manage tomato crop systems.

## 1. Introduction

Tomato (*Solanum lycopersicum* L.) is one of the most economically important cash crops produced worldwide under different environments and latitudes [1]. Recently, the worldwide processing tomato production, suitable to produce specific canning products like peeled tomato, paste and sauce, increased by ~70% [2]. From this point of view, Italy is the most important producer in Europe and the second one in the world [2].

Italian peeled tomatoes, obtained by canning elongated fruits, are well known and required all around the world. The oldest and most famous variety for this tomato-based product is ‘San Marzano’, coming from Southern Italy and showing valuable organoleptic features such as taste, colour, texture and nutritional quality [3,4]. Since 1999, this production is labelled as ‘Pomodoro San Marzano dell’agro sarnese-nocerino’-PDO (Protected Denomination of Origin) by the European Union (http://agricoltura.regione.campania.it/Tipici/pdf/disciplinare_san_marzano_2010.pdf).

Tomato is a source of many nutrients such as citric, ascorbic and other organic acids, sugars, and health-related compounds (carotenoids, flavonoids, and vitamin E). The antioxidant and anticancer properties of tomato and tomato products have been widely proven [5,6,7,8,9,10,11]. Mineral element concentration in tomato fruits may reach 8% of dry matter, for which this vegetable plays a role also in covering the adequate intake (AI) for minerals: one serving of tomato (~200 g) represents 10% of the AI for K for all adults, and about 5–7% of RDA (Recommended Daily Allowance) for P and Mg [12].

Some important technological characteristics like pH, titratable acidity, Hunter colour, and total and soluble solids (expressed as Brix) affect the suitability for tomato processing, and high values of °Brix per ton of marketable yield (per hectare) are requested to achieve great profitability [13].

The presence of nitrate in foods is a serious threat to human health, and in this respect, vegetables contribute, in different diets, more than 80% to daily intake of this unwanted compound [14,15]. Nitrate is converted to nitrite in saliva and along the gastrointestinal tract, causing various complications, including stomach, intestine, bladder and mouth cancers; fetal birth defects; and methemoglobinemia in children [16]. Another implication regards the detinning in canned food by nitrates, when internal epoxy-based coatings are absent. In tomato, for example, high tin concentration is caused by nitrate contained in raw material [17,18].

Nowadays, farmers and researchers have been endeavoring to limit the negative environmental impact due to agricultural practices boosting the increment of crop yield and quality [19,20,21]. In particular, N management strongly affects fruit yield and quality, as well as soil and groundwater sustainability [22,23,24,25].

Plenty of research reported different effects of N fertilization on the overall tomato quality, depending on soil type (N and organic matter content, water availability), cultivation systems (open field or greenhouse), time of application, N form in fertilizers, climatic conditions, and market destination (fresh or processing) [26,27,28,29,30,31,32].

However, nitrogen effects on yield and overall fruit quality of canning genotypes with indeterminate growth habit, still need to be studied in depth. Hence, in this work, the San Marzano tomato landrace was fertilized with different N rates (from 0 to 350 kg ha^−1^) in a typical PDO cultivation area, characterized by high fertility soil conditions. The objective of the study was to establish the N supply optimizing (a) yield, (b) suitability for canning, (c) nutrition (sugar and mineral contents) and (d) sanitary features of raw material (low nitrate content).

## 2. Results

### 2.1. Agronomic Parameters

The effect of the N rate on the agronomic parameters are reported in Table 1 and Table 2. Marketable yield (MY), as the most important yield parameter, was affected by N fertilization and the highest values were displayed by N-125 (52.4 t ha^−1^) and N-200 (53.8 t ha^−1^) which did not significantly differ by N-275 and N-350 treatments. Similar results were also observed for brix yield (BY) (+14%, on average, if compared to the other N supplies). The highest total yield was instead observed for N-200 rate (+29% in respect to N-0); while lower unmarketable yield (UMY) values were found for N-50, N-125; N-275 and N-350 in comparison with the unfertilized control. The highest values of rotten fruits (+37%, respect means of other treatments) and of TSWV-symptomatic fruits by *Tomato Spotted Orthotospovirus* (+18.7%) were noticed at N-350 supply.

The effects of the N fertilization on the biomass production (and its distribution), average fruit weight and N-efficiency are reported in Table 2. N-125 and N-200 treatments displayed the highest values both for fruit dry weight (FDW) and harvest index (HI) (on average + 17% and + 20%, if compared to the other treatments, respectively), while the highest TDW amounts were found at N-275 and N-350 levels (8.7 and 9.0 t per hectare, respectively). N-50 treatment showed the highest value of the average fruit weight (52.6 g), which did not significantly differ from N-0, N-125; N-275 and N-350 supplies. Finally, N-50 also showed the highest value of N-efficiency (+126%, if compared to the means of other treatments), followed by N-125 rate (22.7 kg kg^−1^).

### 2.2. Technological Characteristics and Mineral Compositions

As reported in Table 3, N fertilization did not affect total solids content (TSS), soluble solids content (SSC), titratable acidity (TTA), pH, sugar/TSS (SUG/TSS) ratio or fructose (FRU) content (5.13% fw, 4.75 °Brix, 0.28% fw, 4.47, 53.45%, and 1.29% fw, as means, respectively). Significant increases in glucose (GLU), and then in SUG (GLU + FRU) amounts were instead detected at N-50 (1.40% fw and 1.54% fw, respectively) with respect to unfertilized tomatoes and other N applied rates. Sugar/TSS ratio also reached the highest value at 50 kg ha^−1^ N supply (no significant difference from the others) and TTA/TSS ratio was positively affected by N fertilization from N-0 (4.99%) up to N-200 (5.73%) levels. On the contrary, Hunter colour (a/b) decreased under N supplies (from 2.23 for N-0 to 2.09 for N-275 and N-350). The best values of colour (COL) were found from N-0 to N-200 rate.

As reported in Table 4, nitrate accumulation strongly increased from N-0 (11.33 ppm) to N-350 rate (19.70 ppm) (+73.9%). On the contrary, K content on average decreased as with increasing N supplies (from 2685 ppm of N-0 to 2481 ppm of N-350), and significantly higher values were recorded under N-0 (2685 ppm) and N-125 (2631 ppm) with respect to N-200 (2389 ppm). Mg and phosphates concentration in the fruits were not significantly affected by N applications (117.33 and 364.6 on average, respectively). The highest concentrations of P were detected at low doses of N supplies (0 and 125 kg ha^−1^).

### 2.3. Relationships between Treatments and Evaluated Parameters

**Figure 1:**
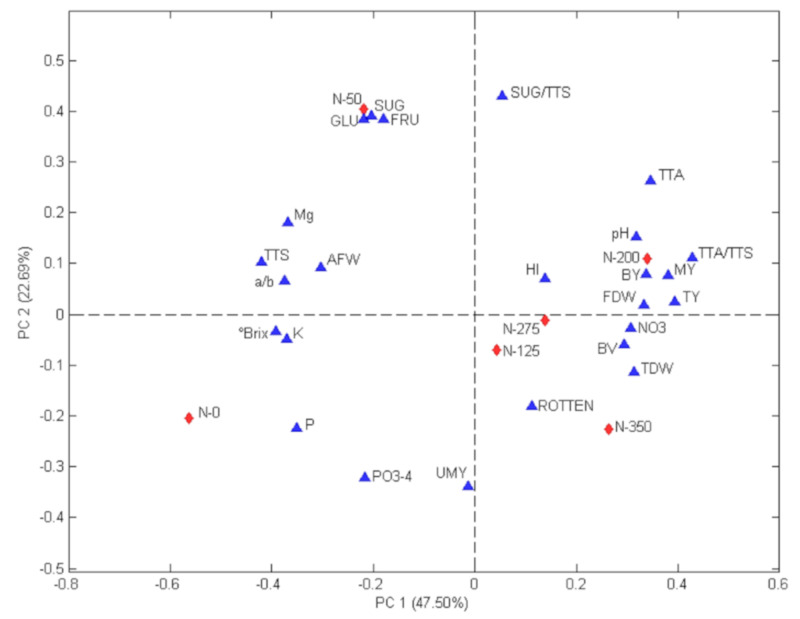
Biplot of Principal Component Analysis results. The assessed treatments (red diamonds) are N-0 = 0 kg N ha^−1^; N-50 = 50 kg N ha^−1^; N-125 = 125 kg N ha^−1^; N-200 = 200 kg N ha^−1^; N-275 = 275 kg N ha^−1^; N-350 = 350 kg N ha^−1^. The studied parameters (blue triangles) are GLU = glucose, FRU = fructose, SUG = GLU + FRU, TTS = total solid content, a/b = fruit colour, AFW = average fruit weight, Brix = soluble solids content, PO3-4 = phosphates, UMY = unmarketable yield, TTA = titratable acidity, HI = harvest index, BY = brix yield, MY = marketable yield, TY = total yield, NO3 = nitrate, BV = symptomatic fruits by TSWV, TDW = total dry weight, FDW = fruit dry weight, ROTTEN = rotten fruits.

The correlations between N rates and the parameters assessed on San Marzano tomato were studied by PCA analysis. Biplot of the PCA models is shown in Figure 1. The contributions of the two first principal components were 47.50% (PC1) and 22.69% (PC2), and their sum explained 70.19% of the total variability. Associations between the N supplies and parameters examined were easily appreciated on biplot, where the first principal component indicated the effects of N rates higher than 50 kg of N ha^−1^. Indeed, N-0 and N-50 treatments were displayed on the negative side and were associated with the majority of the fruit quality parameters, while treatments from 100 to 350 Kg ha^−1^ N were shown on the positive side and were associated with the main agronomic traits. In particular, treatments ranging from N-125 to N-275 were linked with great values of MY, TY, FDW, BY, HI, NO3, pH and TA/TS. On the other hand, N-0 rate was associated with high values of Brix, K and P, and N-50 was related to the best values of GLU, FRU and SUG (GLU + FRU). Finally, the highest N rate (N-350) was associated with the worsening of merceological fruit quality (BV and rotten fruits).

## 3. Discussion

Processing tomato is an important herbaceous crop that requires remarkable agronomic inputs (like fertilizers and irrigation water), which should be appropriately managed, especially in high-fertility conditions, in order to make the tomato production sustainable. Hence, researchers, consultants and farmers should identify, suggest and apply, respectively, the best practices in order to prevent the possible negative environmental impact of farming management.

Different works have investigated the effects of agronomic practices on processing tomato yield and quality as well as on soil microbiota [29,33,34,35], focusing on varieties with determinate growth habit and those cultivated in soils with adequate or low fertility. Conversely, no relevant information is reported on indeterminate varieties for canning purposes cultivated in high soil fertility conditions. The most important issues in crop production regard N management and its soil availability [36,37], which affect vegetative and reproductive phases as well as yield and fruit quality [29,30].

Rainfall and temperature can affect crop yield and quality at different latitudes [38,39,40]. In our study, the growing season was characterized by the lack of rainfall during the period from flowering to harvest. Both the minimum and maximum air temperatures were constantly higher than in the previous 20 years (on average + 2.5 °C for the maximum and + 2.8 °C for the minimum).

In the present study, N-125 and N-200 resulted in higher values of MY, BY and FDW than the other investigated N rates. Moreover, N-125 showed higher values of average fruit weight (AFW) and N-efficiency and lower percent of rotten fruit than N-200 treatments. Our results are in agreement with what was reported for experiments performed under Mediterranean climate conditions [41] as well as the findings of similar researches carried out in California [33]. The percentages of TSWV-symptomatic fruits increased with the N fertilization increase, in agreement with other studies [29,42,43]. Some authors reported that during virus infection, a higher N amount is required to supply to the increasing energy demand necessary to viral RNA and protein synthesis [44].

N rates of 125 and 200 kg ha^−1^ showed the highest values of dry matter allocated to the fruits. These values were almost half of those reported in the literature [45,46], suggesting that the San Marzano landrace was not subjected to any breeding program for HI improvement. Conversely, high dry matter allocation to the fruits is nowadays obtained in modern high-yielding hybrids, both for canning and for fresh market destinations, regardless of vegetative habit and system cultivation (open field or greenhouse conditions). Furthermore, according to the traditional cultivations system, no leaf pruning was made thus promoting dry matter allocation to vegetative organs [46]. The irrigation was carried out by a furrow system, rather than dripline, providing higher supplies than effective Etc-based plant requirements. Other authors reported that the different irrigation systems, adopted over the last 90 years, influenced the processing tomato yield [35,47,48].

The technological parameters and chemical composition of San Marzano fruits were within the ranges reported by Lo Iudice et al. [3], who analyzed seven traditional accessions of the same tomato landrace.

TSS, SSC, TTA, pH and SUG/TSS ratio were not affected by N fertilization consistently, with investigations of some researchers [31,49,50,51] reporting no effect of N supplies from 50 to 250 Kg ha^−1^ on total and soluble solids. Regarding TTA and pH juice, other studies, performed under Southern Mediterranean conditions, also reported no effect of N supplies on these fruit quality attributes [36,43]. Regarding GLU and FRU contents, the effect on N supply seemed poorly explainable for the first sugar, whose highest content was detected at N-50. In any case, our results are of similar magnitude to those reported by Colla et al. [28] and Di Cesare et al. [36] for both carbohydrates. These findings suggested that the most important technological characteristics in tomato are under high genetic control and are poorly affected by some agronomic practices, such as N fertilization. However, among agricultural practices, a large number of studies have shown that irrigation scheduling strongly influences different aspects of tomato quality such as antioxidants, flavor, consistency and other important processing parameters [52,53,54].

The highest value of SUG/TSS ratio was found at N-50, in agreement with Parisi et al. [29].

The decreasing trend of a/b index (COL) was in disagreement with the results of different authors reporting no effect of N fertilization on this quality attribute of processing tomato grown under low–medium soil fertility conditions [29,30,49]. Considering the high correlation between Hunter colour (a/b) and lycopene content in tomato [55], it is possible to suppose no variations of this carotenoid compound under different N rates. Furthermore, Dorais et al. [53] reported that lycopene and other secondary plant metabolites (β-carotene, phenolics and flavonoids) which do not contain N in their molecules are favored under N-limiting conditions, although photosynthetic activity is not reduced, and yield is not decreased. Finally, in our research, no remarkable reduction in plant yield was detected under decreasing N supplies.

Poor information is available in the literature on elemental composition of tomato fruit as an effect of N fertilization. According to De Giorgi et al. [43], no changes in phosphate content were observed under different N supplies (Table 4). Conversely, a decreasing trend of P concentration was found between 0 and 350 Kg ha^−1^, consistently with the results reported by Christou et al. [56]. Regarding the Mg and K accumulation into the fruits, our findings were in accordance with the latter authors. Christou et al. [56] concluded that soil chemical-physical proprieties affect fruit accumulation of these elements to a larger extent than N fertilization; furthermore, irrigation regimes also play a key role.

Finally, our results highlighted that low or no N fertilization resulted in higher concentration of K, Mg, phosphates and P in tomato fruits.

Nitrate content was enhanced by the increase of N supply, in agreement with plenty of research reviewed by Myazaki et al. [57]. It is important to note that, in our experimental conditions, a high nitrate content was also detected in fruits from unfertilized plants. Although fresh-fruit tomato is classified as a very low-nitrate-accumulating vegetable (<200 mg kg^−1^ fw) [58], the canning leads to the concentration of the juice and therefore to the doubling (at least theoretically) of the nitrate content present in the fresh fruit.

Furthermore, the Joint FAO/WHO Expert Committee on Food Additives (JECFA) has recently reviewed the toxicological effects of nitrate and nitrite and established an Acceptable Daily Intake (ADI) of 0–3.7 mg kg^−1^ b.w. (body weight) for nitrate and an ADI of 0–0.07 mg kg^−1^ b.w. for nitrite [59]. According to these recommendations, it appears necessary to reduce the intake of nitrate deriving from canned tomatoes, especially in some food styles (i.e., Mediterranean diet) based on frequent consumption of very high nitrate-accumulating vegetables such as rocket, radish, spinach, lettuce and celery [60].

Another sanitary implication regards the detinning in canned tomatoes caused by nitrates [18]. To prevent the heavy tin dissolving, literature reported the necessity to maintain nitrate–nitrogen concentration in fresh fruit for processing below 3 ppm [61,62]. Future research is required to study how to reduce the fruit nitrate content in processing tomatoes grown in soils with high N total and nitrate concentrations.

## 4. Materials and Methods

### 4.1. Experimental Conditions

**Figure 2:**
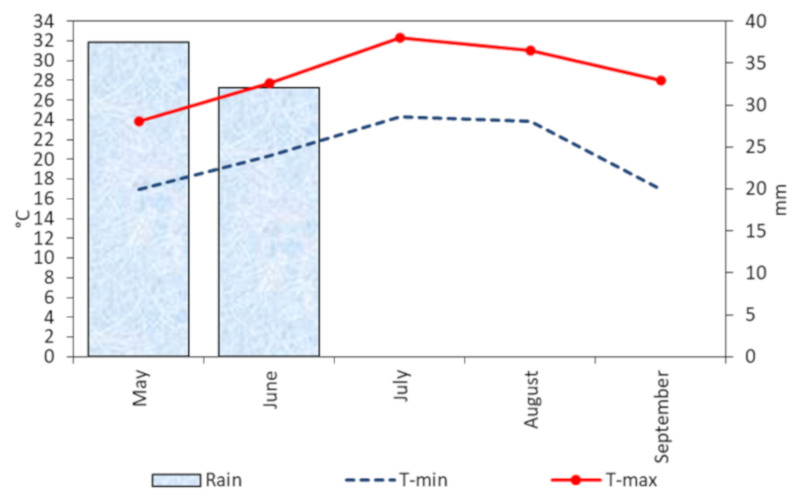
The mean maximum and minimum air temperatures and total rainfall during the cropping cycles (May to September) recorded in the growing season 2015.

The study was carried out during the cropping season 2015 at Angri (Salerno, southern Italy, 40°44′52.8″ N; 14°33′45.3″ E; 29 a.s.l.) on San Marzano tomato landrace (‘SMEC20’ accession). The physical and chemical soil properties were as follows: sand 70.5%, silt 18.0%, clay 11.5%, limestone 4.0%, pH 7.1, organic matter 2.4%, total nitrogen 2.0‰, P_2_O_5_ 137 mg kg^−1^ and K_2_O 682 mg kg^−1^. The climate of this region is typically Mediterranean. A weather station to record the main climatic data was installed in the experimental field. The mean maximum and minimum air temperatures and total rainfall during the cropping cycles (May to September) were 28.6 and 21.5 °C and 69.6 mm, respectively (Figure 2).

### 4.2. Experimental Design, N Application and Crop Management

Seedlings were transplanted on 6 May in single rows with a 0.40 m spacing along the rows, which were 1.0 m apart (2.5 plants per m^2^). K and P requirements, calculated on the basis of soil analysis and plant demands, were supplied prior to transplant: 100 kg ha^−1^ of K_2_O as potassium sulphate and 200 kg ha^−1^ of P_2_O_5_ as triple superphosphate. As for N fertilization, six rates (0, 50, 125, 200, 275 and 350 of N ha^−1^; denoted as N-0, N-50, N-125, N-200, N-275 and N-350) were assessed in a randomized block design with three replications. Each plot measured 6.4 m × 4.0 m and contained 64 plants. The amount of N for each treatment was split into three equal applications (33.3% for each administration) during the crop cycle, at 25 (as ammonium sulphate), 70 and 120 (as ammonium nitrate) days after transplanting. The first two supplies were given before the harvests which (occurred on 5 August and 2 September), and a late N administration was made after the third harvesting (26 September). Plants were furrow-irrigated (once a week with total irrigation volume 4500 m^3^ ha^−1^) and well-watered without any drought stress. Well water, containing ~21 mg L^−1^ of nitrate, was used.

Plant protection and weed control were carried out in accordance with PDO production specifications, including stakes as support and galvanized wires.

### 4.3. Yield Assessment

Productive assessment was performed collecting red-ripe fruits from each of the three harvestings; moreover, at the last one (26 September), unripe fruits were also considered. Yield and its components were recorded (as fruit weight and number) as marketable (ripe), unmarketable (unripe) and rotten fruits. Finally, cumulative values of total (TY), marketable (MY) and unmarketable (UMY) yields, and rotten fruits were finally reported.

At the last fruit harvesting, destructive analyses were carried out (on three plants plot^−1^) by separating fruits from vegetative organs (leaves + stems). Subsequently, these two fractions were weighted and oven-dried at 65 °C until constant weight. Then, fruit dry weight (FDW) and total dry weight (TDW) were reported, and Harvest Index (HI) was also calculated as FDW/TDW × 100.

Furthermore, N-efficiency was calculated according to Ronga et al. [26] and brix t ha^−1^ (BY) was obtained by multiplying the hectare marketable yield by Brix and dividing the result by 100. Average fruit yield (AFW) was obtained by dividing MY and total number of mature fruits collected on the three harvestings.

In a sample of 100 mature fruits (randomly chosen by each plot), fruits infected by TSWV—*Tomato Spotted Orthotospovirus*) and showing typical symptoms as chlorotic blotches and ringspots were also counted.

### 4.4. Fruit Quality Analyses

Fruits subjected to quality analysis were those collected in the most fruitful harvest (2 September). Well-ripened fruits (2500 g per plot), were washed and dried, and then sliced and homogenized in a Waring blender (2 L capacity; Model HGB140, CA, USA) for 1 min.

#### 4.4.1. Technological Characteristics

Titratable acidity (TTA) (expressed as g of citric acid L^−1^ juice) and pH were determined using pH-Matic 23 titroprocessor equipped with pH electrode with a temperature sensor (model 5011T) (Crison Instruments, Barcelona, Spain). Soluble solids content (SSC) was instead measured using a digital refractometer (Refracto 30PX, Mettler-Toledo, Novate Milanese, IT), and the results were expressed as Brix on 100 g of fresh weight (fw). The determination of the total solids (TSS) was carried out by drying 10 g of homogenized sample in a stove at 70 °C until the complete elimination of water.

The colour (COL) was measured using a Minolta CR 300 Chroma portable colourimeter (Minolta Co., Osaka, Japan) with C illuminant. The colourimeter was calibrated with a white standard calibration plate (Y = 93.9, x = 0.3134, y = 0.3208) before use. Colour was measured on the equatorial region of ten fruits/plot and expressed as a*/b* index.

#### 4.4.2. Sugar Analysis

Glucose (GLU) and fructose (FRU) contents were determined by HPLC (mod. 600 E liquid chromatograph) equipped with a Lichrosorb-NH2 (10 µm) (Merck, Darmstadt, Germany) and acetonitrile/water mixture (80: 20; *v/v*) as eluent. As conductivity detector, the model 431 by Waters (Dublin, Ireland) was used [49]. Total sugars (SUG), as the sum of GLU and FRU, were also reported.

SUG/TTA and total SUG/SSC ratios were also calculated as the quality index of tomato fruits influencing their acceptability [63].

#### 4.4.3. Mineral Composition

##### Sample Preparation

For mineral determination, 10.0 ± 1.0 g of homogenized paste were placed in a muffle furnace at 550 ± 10 °C for 24 h until complete incineration [64]. Then, the incinerated samples, cooled in a desiccator until room temperature, were recovered and boiled in 10 mL of a hydrochloric acid solution in water (1 + 3) and, after cooling, were transferred by filtration in 50.0 mL volumetric flasks.

##### Analytical Determinations

Magnesium content was determined by atomic absorption spectrometry in a Perkin-Elmer spectrophotometer Analyst 200 (ILC, Lisbon, Portugal) using an air-acetylene flame.

Potassium content was measured by a Jenway PFP7 flame photometer (Dunmow, UK) using an air-propane flame [64,65]. Phosphorus content was determined by a BioMate™ 160 UV-Vis Spectrophotometer (Waltham, MA, USA) in transmittance mode at λ = 720 nm, using ultrapure water to adjust transmittance to 100% and a 2 μg/mL phosphorus solution as a standard [64,65].

Nitrate and phosphate contents were determined by a Waters (Dublin, Ireland) HPLC (mod. 600) equipped with an IC-PAK TM Anion column (100 mm × 4.6 mm) and a conductivity detector (model 431) using a borate /gluconate buffer at pH 8.5 as eluent.

Finally, the total N concentration of the fruit samples was determined by the Kjeldahl method [66], following mineralization with sulphuric acid (96%).

### 4.5. Data Analysis

All recorded data were subjected to analysis of variance (one-way ANOVA). Means were separated using a Tukey test, when the F test of ANOVA for treatment was significant at *p* < 0.05. In addition, all collected data during the experiment, apart from N-efficiency, were analyzed by the Principal Component Analysis (PCA) model [67,68] to study the relationships between the analyzed objects and the original variables, and a biplot graph was used. For statistical analysis, GENSTAT 17th software package (VSN International, Hemel Hempstead, UK) was used.

## 5. Conclusions

Our results displayed that N fertilization, as one of the most yield-impacting factors on tomato crops, is challenging in high fertility soil conditions. Therefore, knowledge of N requirements in relation to soil fertility and cultivar genetic background is necessary to optimize N supply, i.e., targeting high yield and fruit quality, as well as the tomato crop sustainability.

Indeed, in our study, 125–200 Kg ha^−1^ of N resulted in better agronomic performances than higher N rates. The optimal N rate (125 kg ha^−1^) led to the high N-efficiency, as well as to the best nutritional (K and P concentrations) and healthy fruit quality (reduction of rotten fruits). N rates exceeding 125 kg ha^−1^ negatively impacted agronomic performance and some fruit quality attributes and compromised the sustainability of San Marzano landrace cultivated in high fertility soil conditions.

## Figures and Tables

**Table 1:** Effects of nitrogen supply (N-0 = 0 kg N ha^−1^; N-50 = 50 kg N ha^−1^; N-125 = 125 kg N ha^−1^; N-200 = 200 kg N ha^−1^; N-275 = 275 kg N ha^−1^; N-350 = 350 kg N ha^−1^) on yield and its components. MY = marketable yield, TY = total yield, UMY = unmarketable yield, BY = brix yield, TSWV = symptomatic fruits by *Tomato Spotted Orthotospovirus*.

Treatments	MY (t ha^−1^)		TY (t ha^−1^)		UMY (t ha^−1^)		ROTTEN (Fruit no.)		BY (t ha^−1^)		TSWV (Fruit no.)	
N-0	35.3	c	55.7	b	11.7	a	8.7	b	1.7	b	12.0	b
N-50	43.0	bc	59.4	b	8.2	b	8.2	bc	2.1	b	15.0	ab
N-125	52.4	a	66.2	ab	7.1	b	6.8	c	2.5	a	15.3	ab
N-200	53.8	a	71.8	a	9.4	ab	8.5	b	2.5	a	14.0	ab
N-275	45.9	ab	62.0	ab	8.0	b	8.1	bc	2.1	ab	17.7	ab
N-350	46.2	ab	64.8	ab	6.8	b	11.9	a	2.1	ab	18.7	a
Average	46.1		63.3		8.5		8.7		2.2		15.5	
*p*-value	*		*		*		*		*		*	

* statistically significant at *p* ≤ 0.05. Different letters within each column indicate significant differences according to Tuckey’s test (*p* ≤ 0.05).

**Table 2:** Effects of nitrogen supply (N-0 = 0 kg N ha^−1^; N-50 = 50 kg N ha^−1^; N-125 = 125 kg N ha^−1^; N-200 = 200 kg N ha^−1^; N-275 = 275 kg N ha^−1^; N-350 = 350 kg N ha^−1^) on fruit dry weight (FDW), total dry weight (TDW), harvest index (HI), average fruit weight (AFW) and N-efficiency.

Treatments	FDW (t ha^−1^)		TDW (t ha^−1^)		HI		AFW (g)		N-Efficiency (kg kg^−1^)	
N-0	2.0	c	7.1	b	0.28	b	51.9	ab	-	
N-50	2.2	bc	7.6	b	0.28	b	52.6	a	43.3	a
N-125	2.8	a	7.6	b	0.38	a	51.3	ab	22.7	b
N-200	2.8	a	7.8	b	0.36	a	50.1	b	14.2	c
N-275	2.5	ab	8.7	a	0.29	b	51.6	ab	9.2	d
N-350	2.4	bc	9.0	a	0.26	b	51.6	ab	6.6	d
Average	2.4		8.0		0.31		51.5		19.2	
*p*-value	*		*		*		*		*	

* statistically significant at *p* ≤ 0.05. Different letters within each column indicate significant differences according to Tuckey’s test (*p* ≤ 0.05).

**Table 3:** Effects of nitrogen fertilization (N-0 = 0 kg N ha^−1^; N-50 = 50 kg N ha^−1^; N-125 = 125 kg N ha^−1^; N-200 = 200 kg N ha^−1^; N-275 = 275 kg N ha^−1^; N-350 = 350 kg N ha^−1^) on quality attributes of tomato fruits.

Treatment	TSS % fw		SSC Brix		TTA % Citric Acid		TTA/TTS %		GLU % fw		FRU % fw		SUG % fw		SUG/TSS %		pH		COL a/b	
N-0	5.28	a	4.92	a	0.26	a	4.99	b	1.29	b	1.45	a	2.74	ab	52.0	a	4.40	a	2.23	a
N-50	5.27	a	4.79	a	0.29	a	5.44	ab	1.40	a	1.54	a	2.94	a	55.8	a	4.47	a	2.18	ab
N-125	5.13	a	4.84	a	0.28	a	5.46	ab	1.27	b	1.40	a	2.67	ab	52.5	a	4.52	a	2.15	ab
N-200	5.01	a	4.65	a	0.29	a	5.73	a	1.28	b	1.45	a	2.73	ab	54.5	a	4.50	a	2.15	ab
N-275	5.04	a	4.64	a	0.28	a	5.63	ab	1.29	b	1.43	a	2.72	ab	54.0	a	4.47	a	2.09	b
N-350	5.05	a	4.64	a	0.28	a	5.64	ab	1.23	b	1.41	a	2.63	b	51.9	a	4.46	a	2.09	b
Average	5.13		4.75		0.28		5.48		1.29		1.45		2.74		53.5		4.47		2.15	
*p*-value	NS		NS		NS		*		*		NS		*		NS		NS		*	

* statistically significant at *p* ≤ 0.05. TSS = total solids content, SSC = soluble solids content, TTA = titratable acidity, FRU = fructose, GLU = glucose, SUG = sugar, COL = Hunter colour. NS, non-significant or significant at *p* ≤ 0.05. Different letters within each column indicate significant differences according to Tuckey’s test (*p* ≤ 0.05).

**Table 4:** Effects of nitrogen fertilization (N-0 = 0 kg N ha^−1^; N-50 = 50 kg N ha^−1^; N-125 = 125 kg N ha^−1^; N-200 = 200 kg N ha^−1^; N-275 = 275 kg N ha^−1^; N-350 = 350 kg N ha^−1^) on chemical composition of tomato fruits.

Treatment	Nitrate (ppm)		K (ppm)		Mg (ppm)		Phosphates (ppm)		P (ppm)	
N-0	11.33	c	2685.0	a	122.2	a	385.7	a	137.8	a
N-50	14.93	b	2605.0	ab	121.5	a	346.3	a	111.8	b
N-125	13.07	bc	2631.0	a	118.5	a	382.9	a	142.5	a
N-200	15.07	b	2389.0	b	115.9	a	345.2	a	96.5	b
N-275	14.93	b	2590.0	ab	115.8	a	365.1	a	105.3	b
N-350	19.70	a	2481.0	ab	110.1	a	362.2	a	114.8	b
Average	14.84		2563.5		117.3		364.6		118.1	
*p*-value	*		*		NS		NS		*	

NS, * non-significant or significant at *p* ≤ 0.05. Different letters within each column indicate significant differences according to Tuckey’s test (*p* ≤ 0.05).
